# Predictors and kinetics of ATO-induced leukocytosis in acute promyelocytic leukemia patients: a retrospective study

**DOI:** 10.1007/s12672-026-05155-1

**Published:** 2026-05-09

**Authors:** Hong Wei, Zhuo Zhang, Xueli Liu, Qian Zhou, Xin Zhang, Ying Dong, Siqi Zhai, Qian Zhang, Meijuan Sui

**Affiliations:** 1 Department of Hematology, The First Affiliated Hospital of Harbin Medical University, Harbin, China; 2 Department of Scientific Research and Education, Southern University of Science and Technology Hospital, Shenzhen, China; 3 Central Laboratory of the First Affiliated Hospital of Harbin Medical University, 23 Youzheng Street, Nangang District, Harbin, 150001 Heilongjiang China; 4 Department of Hematology, Inner Mongolia People’s Hospital, Hohhot, China; 5 Department of Laboratory Diagnostics, First Affiliated Hospital of Harbin Medical University, Harbin, China; 6 Key Laboratory of Hepatosplenic Surgery, Ministry of Education, Harbin, China

**Keywords:** Acute promyelocytic leukemia, Arsenic trioxide, Kinetic feature, Leukocyte, Predictors

## Abstract

**Background:**

Although Arsenic trioxide (ATO) has revolutionized acute promyelocytic leukaemia (APL) from a uniformly fatal disease to one of the most curable leukemias, leukocytosis is a common life-threatening side effect and is associated with inferior results. This study aimed to describe leukocyte proliferation kinetics and explore predictors of leukocytosis among APL patients in order to guide individualized application of ATO.

**Results:**

In this retrospective study of 100 patients, the incidence of leukocytosis in the single drug ATO group was 74.5%, while that in the ATO- chemotherapy group was 93.9% (*P* = 0.018). The dynamic changes in leukocytosis showed a higher WBC count in the ATO-chemotherapy group. The median time to leukocytosis was the 9th day (IQR, 6.00–14.00). The duration of leukocytosis was shorter in the ATO single agent group. The uni-variate analysis and multi-variable analysis showed that the same independent prognostic factor was *the time to double WBC* for leukocytosis for all patients (OR 11.459, 95% CI, 2.121–61.897) and ATO single agent patients (OR 16.603, 95% CI, 1.635-168.635). The area under the ROC curve was 0.835 (95% CI, 0.700-0.971) for all APL patients and 0.822 (95% CI, 0.675–0.969) for *single ATO-induced leukocytosis*.

**Conclusions:**

The kinetics of leukocytosis induced by single-agent ATO and ATO-chemotherapy were different. *The time to double WBC≤7th day* was identified as an independent poor prognostic factor for single ATO-induced leukocytosis in APL patients.

**Supplementary Information:**

The online version contains supplementary material available at 10.1007/s12672-026-05155-1.

## Background

Arsenic trioxide (As_2_O_3_, ATO), a primary treatment for acute promyelocytic leukemia (APL), has been shown to increase complete remission (CR) [1, 2],, even in relapsed patients (91% of the patients entered complete hematological remission) [3]. Due to the high efficacy of ATO, APL has been revolutionized from a uniformly fatal disease to one of the most curable leukemias [4]. In APL cells, the anti-tumor activity of ATO is related to the induction of differentiation when a low dose is used and apoptosis when a high dose is used [5, 6]. Leukocytosis is a common side effect, and there is a high possibility of differentiation syndrome (DS), a life-threatening complication [7]. There is a study showing that the analysis characteristics of leukemia cells improves risk stratification and prediction of chemotherapy response in patients [8]. If patients lack sufficient early clinical intervention, the mortality rate is as high as 26.7% [9]. Several studies have found that leukocytosis especially progressive leukocytosis, plays an important role in the development of DS, particularly among patients with a peak white blood cell (WBC) count > 10 × 10^9^/L [9–11]. In patients developing leukocytosis during ATO-based induction therapy, the risk of early complications is substantially increased, including differentiation syndrome and early death [9, 12].While leukocytosis has been consistently associated with poor short-term outcomes such as differentiation syndrome and early death in APL, its prognostic impact on long-term event-free survival in ATO-treated populations may be less pronounced than previously recognized [13–15]. Therefore, it may be important to study the leukocyte proliferation kinetics features during ATO-based induction therapy for the prediction and prevention of leukocytosis and DS in de novo acute promyelocytic leukemia patients. However, few studies have explored the leukocyte proliferation kinetics and predictors of leukocytosis in APL patients treated with ATO as a front-line treatment [16, 17]. In this retrospective study, We aimed to describe leukocyte proliferation kinetics and clinical features and explore the incidence and predictors of ATO-induced leukocytosis in APL patients. Recognizing the risk factors for leukocytosis and providing better prophylactic treatment are important in clinical practice.

## Methods

### Patient selection

**Fig. 1:**
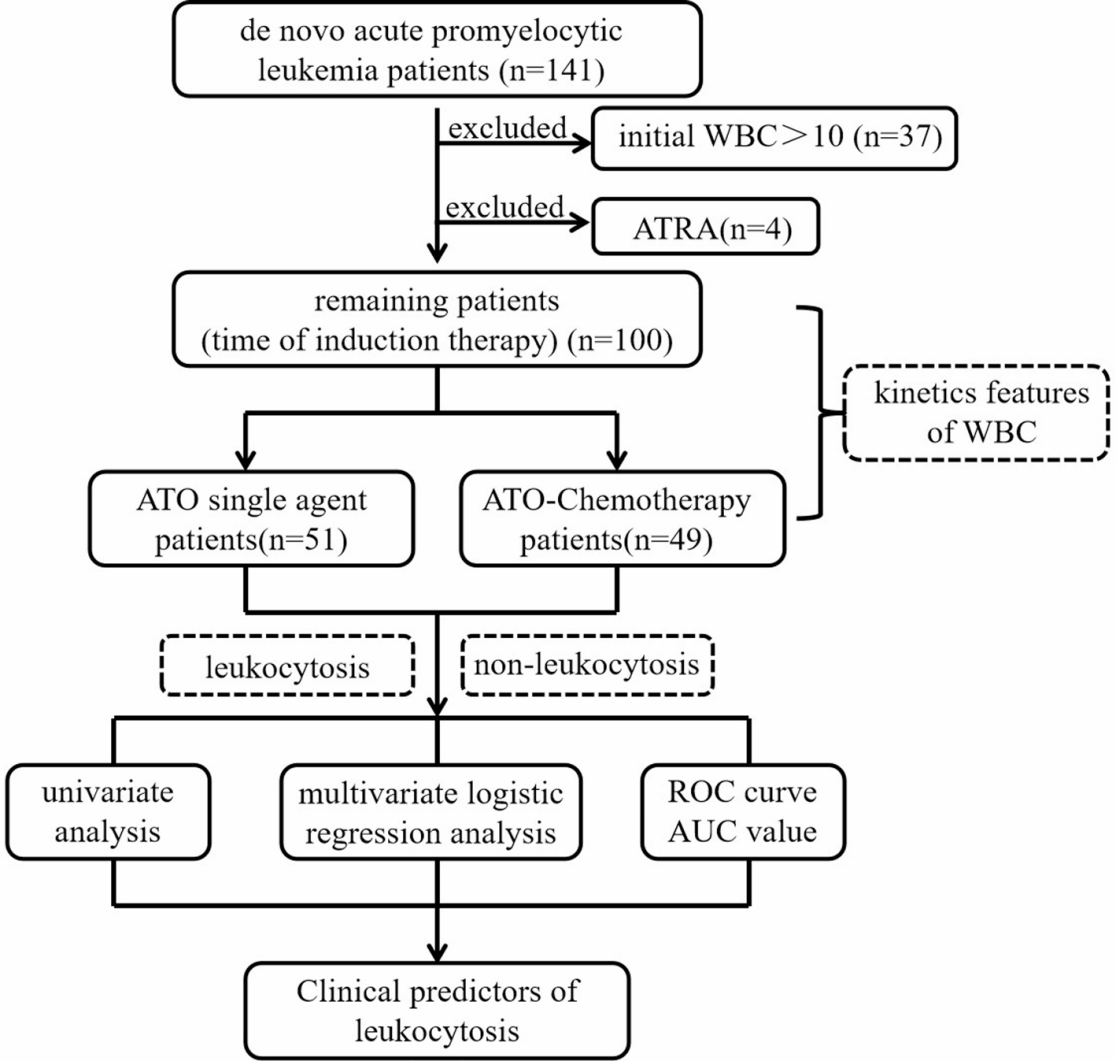
Flow chart of the patient selection process in the schedule. The enrolled patients received ATO for 4 weeks of induction therapy. Chi-square tests and multivariate logistic regression analysis were used to examine ATO-induced leukocytosis. 37 patients were excluded from the study because their initial WBC count >10 × 10^9^ /L, 4 patients were excluded from the study because their treatment of ATRA, and thus, they could not be evaluated for the leukocytosis of As_2_O_3_. *ATO* arsenic trioxide, *WBC* white blood cell count, *ATRA* All-trans retinoic acid, *ROC* Receiver operating characteristic, *AUC* area under the ROC curve

The 100 patients in this retrospective study were newly diagnosed APL patients from October 2012 to September 2019 in our hospital (Fig. 1). All patients were treated by the hematology physicians of the hospital. The ethics committee confirmed that this study was exempt from the need for informed consent due to the retrospectively observational nature of the study. The study did not include confidential data and interventions and patient data were anonymized. Thus, the study did not include confidential data and interventions. The inclusion criteria were a, the diagnosis was confirmed by the presence of t (15; 17) and/or the PML/RARα fusion gene; b, the APL patients were newly diagnosed and first treated with ATO-based induction therapy; c, patients with initial WBC counts (WBC count at diagnosis) in peripheral blood ≤ 10 × 10^9^/L, and d, all patients had monitoring with complete blood counts (CBCs) during the whole course of ATO-based induction therapy. The exclusion criteria were as follows: a, abnormal renal function, liver function or electrocardiographic findings; b, patients with initial WBC counts (WBC count at diagnosis) in peripheral blood > 10 × 10^9^/L; and c, patients who could not be evaluated because personal reasons precluded them from receiving ATO treatment. The patient selection process in the schedule as Fig. 1. All patients were newly diagnosed (de novo) APL patients receiving first-line ATO-based induction therapy. No relapsed or refractory patients were included. The choice between ATO single-agent and ATO-chemotherapy regimens was based on physician discretion and contemporary institutional guidelines for APL management in China [18], primarily considering the patient’s age, performance status, and comorbidities.

### Definition and clinical indicators of ATO-induced leukocytosis

For all newly diagnosed APL patients, leukocytosis was defined as a peripheral blood WBC count greater than 10 × 10^9^/L during ATO-based induction therapy [16–20]. The basic indicators or laboratory parameters were collected from medical records for further analysis according to clinical experience and previous relevant literature, and all indicators were noninvasive, easy to observe, and included the following: age, sex, initial WBC count, initial platelet count (Plt), initial hemoglobin (Hb), initial fibrinogen (FIB) and initial liver enzymes (ALT, AST and GGT). The evaluation of leukocytosis includes *initial WBC counts*, *the time to double WBC* (the needed time for “double the initial WBC count”), *the time to leukocytosis* (the onset of leukocytosis), *the time to peak WBC* (the needed time for “peak WBC”), *peak WBC*, and ΔWBC. Liver enzymes (ALT, AST, and GGT) were included given that the liver is the primary site of ATO metabolism and hepatic function may modulate ATO bioavailability and differentiation-inducing effects; furthermore, GGT has been shown to positively correlate with leukocyte count changes in APL patients during ATO treatment [21, 22].

### Treatment protocol and the proliferation kinetics of WBCs

The ATO was administered as continuous slow-rate infusion in ATO single-agent patients, in which the As_2_O_3_ solution (10 mg/10 mL) was supplied by Harbin Yida Pharmaceutical Company, dissolved in 500 mL 5% dextrose and administered daily at a dose of 0.20 mg/kg (a maximum daily dose of 10 mg) and the total ATO dose was infused intravenously over the course of > 18 h. The ATO-chemotherapy patients were treated with conventional As_2_O_3_ therapy (0.16 mg/kg/d) and intravenous injection of chemotherapy drugs at the same time. The chemotherapy drugs are daunorubicin (40 mg/m^2^/d) for 3 days, cytarabine (100mg/m^2^/d) for 5 days or homoharringtonine (2 mg/m^2^/d) for 7 days. Hydroxyurea (1.5–2.5 g/6 h) was used to control hyperleukocytosis adjusted according to WBC dosage during induction therapy while discontinued when WBC count dropped below 10 × 10^9^/L as per APL0406-like recommendations [1, 19] in ATO-chemotherapy patients. In the ATO single-agent group, no cytoreductive agents were administered when leukocytosis developed. Patients were closely monitored with daily complete blood counts and clinical assessment; supportive care was provided as clinically indicated, and no life-threatening complications requiring emergency intervention occurred in this group. In the ATO-chemotherapy group, leukocytosis was managed primarily through the cytoreductive effect of concurrent chemotherapy, with hydroxyurea added when necessary as described above. Therefore, leukocytosis management was not identical between the two groups. Peripheral blood CBCs were performed every day during the 1st week and approximately every other two days starting from the 2nd week during the treatment and the recording times were days 1–7, 10, 13, 17, 18, 22, 25, and 28 of ATO induction therapy. We analyzed the extent of leukocytosis and the difference in leukocyte kinetics of APL patients. The leukocyte kinetics recordings included initial WBC, peak WBC, ΔWBC (WBC changes between peak WBC and initial WBC), *the time to double WBC* (the needed time for double the initial WBC count), *the time to leukocytosis* (the onset of leukocytosis), and *the time to peak WBC* (the needed time for “the peak WBC”).

### Statistical analysis

All data were analyzed using SPSS 27.0 software and Graph Pad Prism 9.0. For the numerical variables that were normally distributed, the mean (standard deviation) was used for statistical description, and the t test was used for comparison between the groups. Otherwise, the median (IQR, P_25_, P_75_) was used for description, and nonparametric test (Mann‒Whitney U test) was used to compare two unmatched groups. Categorical variables are described as frequencies (percentages) and were analyzed by the chi-square test or Fisher’s exact test (*n* < 5). Spearman correlation was used to quantify the association among leukocytosis variables. Multivariate logistic regression was used to determine the independent association between variables and leukocytosis. Receiver operating characteristic (ROC) curve analysis and the area under the ROC curve (AUC) were used to evaluate the ability of the prediction models to screen for leukocytosis. Two-tailed *P* values are shown for all analyses and a *P* value of less than 0.05 was considered statistically significant.

### Study design, sample size and ethics

This is a retrospective cohort study. As all data were collected from existing medical records, no a priori power calculation was performed; the sample size was determined by the number of consecutive patients who met the inclusion criteria during the study period. The study protocol was approved by the Ethics Committee of the First Affiliated Hospital of Harbin Medical University and was exempt from informed consent owing to its observational retrospective design. No large language models (LLMs) were used in the preparation of this manuscript.

## Results

### Baseline clinical characteristics of the study populations

Between October 2012 and September 2019, 141 de novo APL patients were included according to the inclusion criteria, 41 were excluded from the study. First, 4 patients were excluded from the study because of their treatment with ATRA. Second, 37 patients who met the exclusion criteria (*initial WBC counts* > 10 × 10^9^/L) were excluded. In total, data on leukocytosis were available for only 100/141 (70.92%) patients in this retrospective study. The main clinical characteristics of these patients were collected and are summarized in Table 1.

**Table 1:** Baseline characteristics on admission of APL patients with ATO treatment

Characteristic	Total patients (*n* = 100)	ATO single agent group (*n* = 51)	ATO-chemotherapy group (*n* = 49)	*P*
Age, years	40.50 (31.25-52.00)	45.00 (31.00–52.00)	37.00 (31.50–47.00)	0.061
Sex, n (%)				0.343
Female	51 (51.00%)	25 (47.17%)	26 (53.06%)	
Male	49 (49.00%)	26 (50.98%)	23 (46.94%)	
Initial WBC count, ×10^9^/L	1.54 (0.92–2.98)	1.53 (0.93–3.31)	1.73 (0.89–2.98)	0.449
Initial hemoglobin level, g/L	77.74 (60.38–94.21)	81.95 (65.61–99.40)	76.01 (57.17–89.76)	0.035^*^
Initial platelet count, ×10^9^/L	23.93 (15.60-40.02)	26.28 (15.99–65.30)	23.00 (15.10-33.05)	0.047^*^
Initial fibrinogen level, g/L	1.40 (0.92–2.50)	1.60 (1.20–2.56)	1.19 (0.90–2.07)	0.030^*^
Initial GGT, U/L	27.00 (17.2–42.5)	26.95 (17.05–44.8)	27.40 (18.00-35.80)	0.065
Initial AST, U/L	21.40 (15.10–29.00)	24.90 (13.48–36.60)	20.2 (15.25-31.00)	0.383
Initial ALT, U/L	22.00 (13.50–36.00)	21.70 (14.90-27.13)	18.40 (13.85–35.50)	0.4665
The time to double WBC, day	7.00th (5.000th -10.00th )	8.50th (4.25th -13.75th )	6 .00th (5.00th -8.00th )	0.0195^*^
The time to leukocytosis, day	9.00th (6.00th -13.00th )	10.00th (6.00th -15.00th )	9.00th (6.00th -13.00th )	0.1425
The peak WBC, ×10^9^/L	21.68 (12.66–36.15)	20.72 (9.70-35.72)	21.79 (16.11–39.58)	0.033^*^
The time to peak WBC, day	16.00th (11.00th -20.00th )	16.00th (11.00th -22.00th )	15.00th (10.00th -19.00th )	0.091
ΔWBC, ×10^9^/L	19.57 (9.28–33.22)	17.90 (7.29–31.85)	19.86 (13.77–35.73)	0.0325^*^

Data are n (%) or median (IQR); bold values and ^*^are statistically significant (P < 0.05)

ALT: alanine aminotransferase; AST: aspartate aminotransferase; GGT: gamma-glutamyltransferase; WBC: white blood cell count; ATO: arsenic trioxide. ΔWBC WBC changes between the peak WBC and initial WBC before ATO treatment

### Kinetic features and characteristics of leukocytosis

Dynamic recording of WBC was performed during the whole course of ATO-based induction therapy, in which CBCs were taken every day during the 1st week, and CBCs were performed every three days starting from the 2nd week during the treatment. The results were statistically analyzed as follows.

**Fig. 2:**
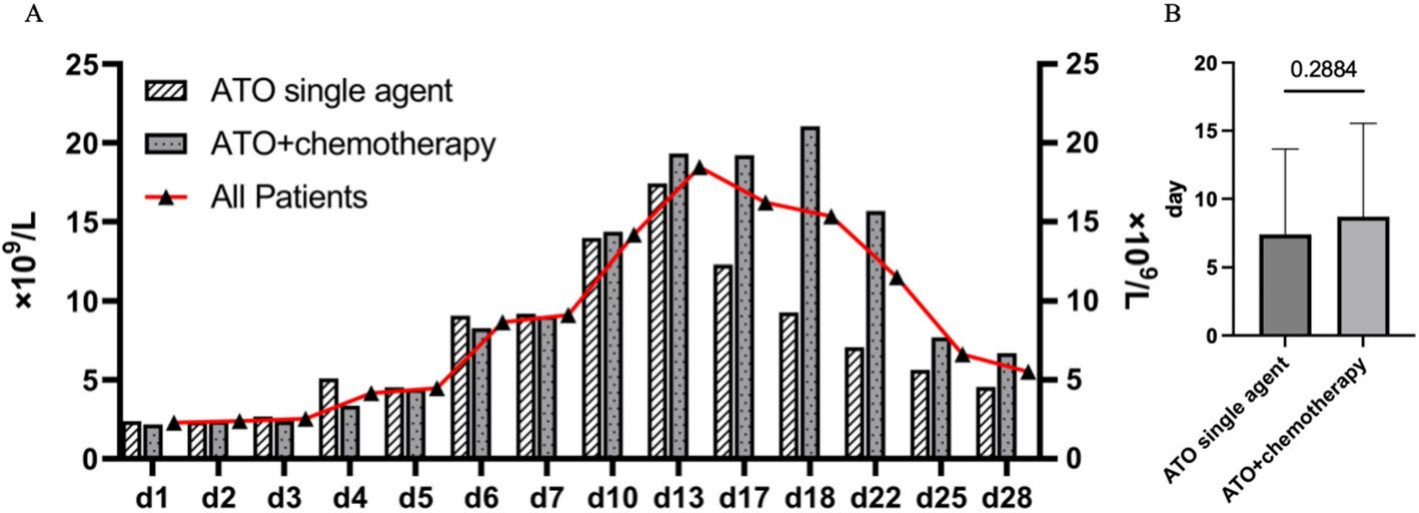
The kinetics of leukocyte proliferation and duration of leukocytosis in APL patients during ATO induction therapy. **A** WBC kinetics during ATO-based induction therapy; WBC, white blood cell count, median (IQR). **B** Duration of leukocytosis (WBC > 10 × 10⁹/L) in patients who developed leukocytosis, shown as mean ± SD; Mann-Whitney U test, *P* = 0.2884. *WBC* white blood cell count, median (IQR), *ATO* arsenic trioxide, *APL* acute promyelocytic leukemia

Absolute leukocyte counts showed similar kinetic changes in “ATO single agent patients” and ATO-chemotherapy patients. The patients developed leukocytosis at some point during induction therapy with ATO. The peak WBC was around the 13th day among all patients and ATO single agent patients earlier than in the ATO-chemotherapy group. The duration of leukocytosis (WBC > 10 × 10⁹/L) was longer in “all patients” and in the ATO-chemotherapy group than in the ATO single agent group. The duration of leukocytosis was compared between the two groups, excluding patients whose leukocytosis had not resolved within the 28-day observation window (ATO single agent, *n* = 47; ATO+chemotherapy, *n* = 43). The mean duration was 7.23 ± 6.30 days in the ATO single agent group and 8.70 ± 6.84 days in the ATO+chemotherapy group, with no statistically significant difference observed between the two groups (Mann-Whitney U test, Fig. 2B, *P* = 0.2884).

**Fig. 3:**
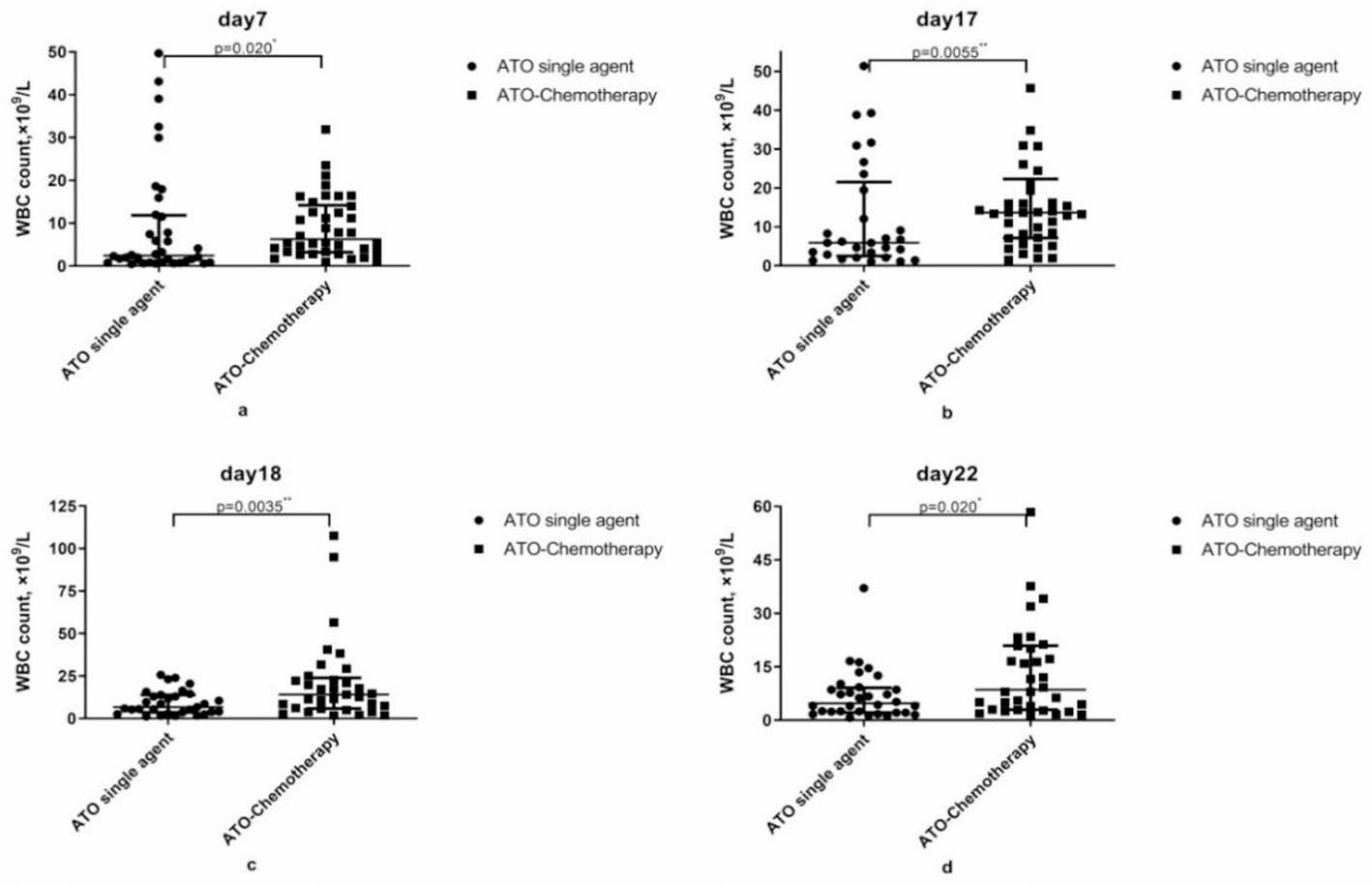
The median WBC counts at some points during ATO-based induction therapy. *ATO* arsenic trioxide, *APL* acute promyelocytic leukemia. Statistical comparisons were performed at each pre-specified time point using the Mann-Whitney U test; only time points with statistically significant differences between the two groups (*P* < 0.05) are marked with asterisks (*)

The median WBC count showed significant differences at some points between the two treatments during induction therapy (Fig. 3). The median WBC counts in patients with ATO single agent were lower than in patients with ATO-chemotherapy (Fig. 3) (12.31 × 10^9^/L vs. 19.217 × 10^9^/L, day 17; 9.285 × 10^9^/Lvs. 21.05 × 10^9^/L, day 18 and 7.075 × 10^9^/L vs. 15.677 × 10^9^/L, day 22, respectively). The largest difference occurred on the 18th day.

**Fig. 4:**
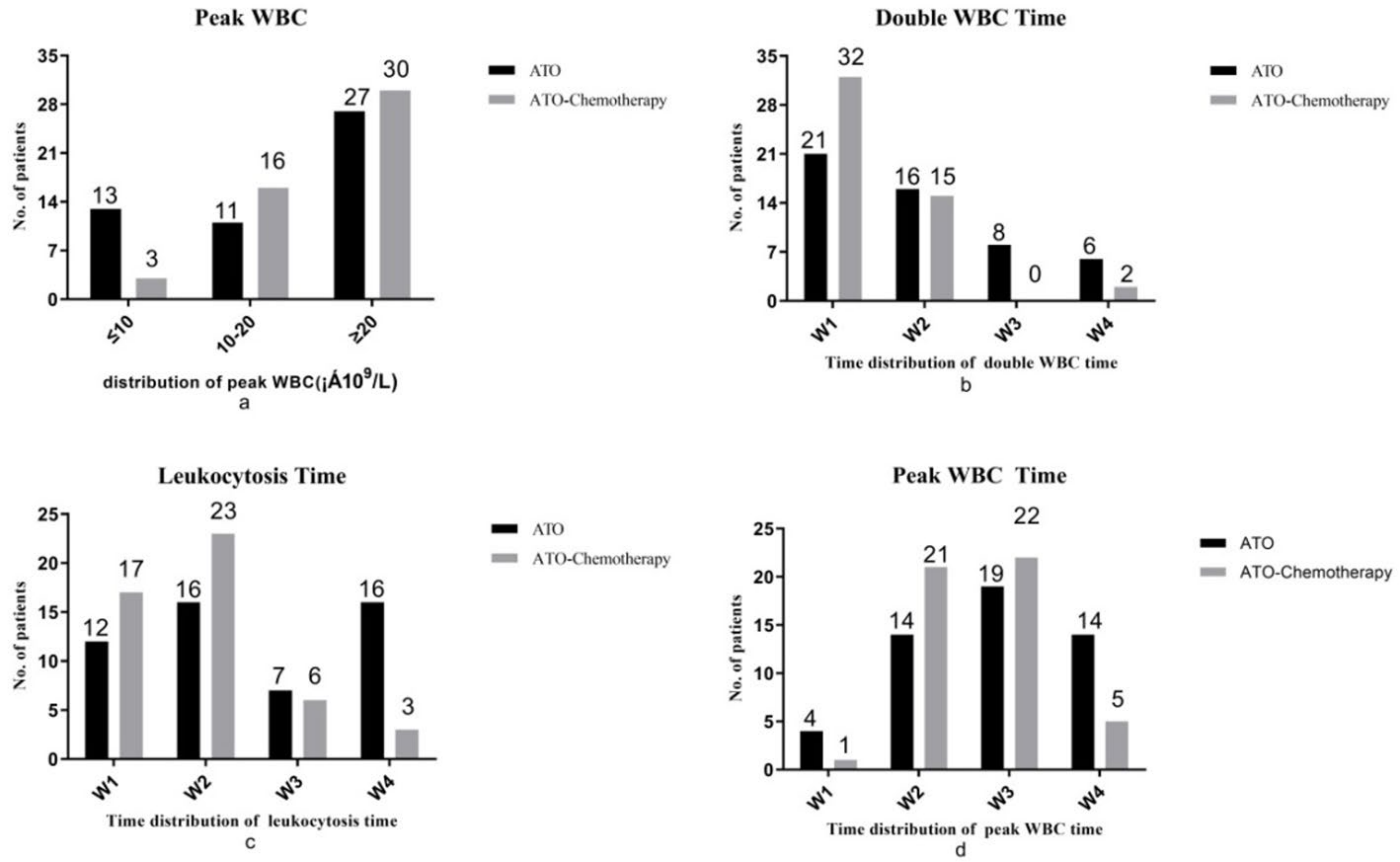
The temporal distribution and intensity of leukocytosis in APL patients. The intensity of *peak WBC* (**A**); the temporal distribution of double WBC time (**B**), leukocytosis time (**C**), and *peak WBC* time (**D**). *W1*, the 1st week; *W2*, the 2nd week; *W3*, the 3rd week; *W4*, the 4th week during ATO treatment; *ATO* arsenic trioxide, *APL* acute promyelocytic leukemia

The intensity of “the peak WBC ≥ 20 × 10^9^/L” accounted for the majority which was more than half in ATO single agent and ATO-chemotherapy groups (Fig. 4a). *The time to double WBC* occurred in the first 2 weeks, accounted for the majority of patients in both groups and the temporal distribution of double WBC time occurred most frequently in the 1st week (Fig. 4b). The trends in the temporal distribution of time to leukocytosis were similar to those of *the time to double WBC* but occurred most frequently in the 2nd week of induction therapy (Fig. 4c). *The time to peak WBC* occurred in the 3rd week of induction therapy and accounted for the majority of two groups (Fig. 4d).

### Correlations of leukocytosis indicators in the study populations

Spearman’s correlation analysis of leukocytosis indicators was applied in all group patients. The results showed that there were close relationships among the indicators, among which the correlation between ΔWBC and peak WBC was the most significant.

**Fig. 5:**
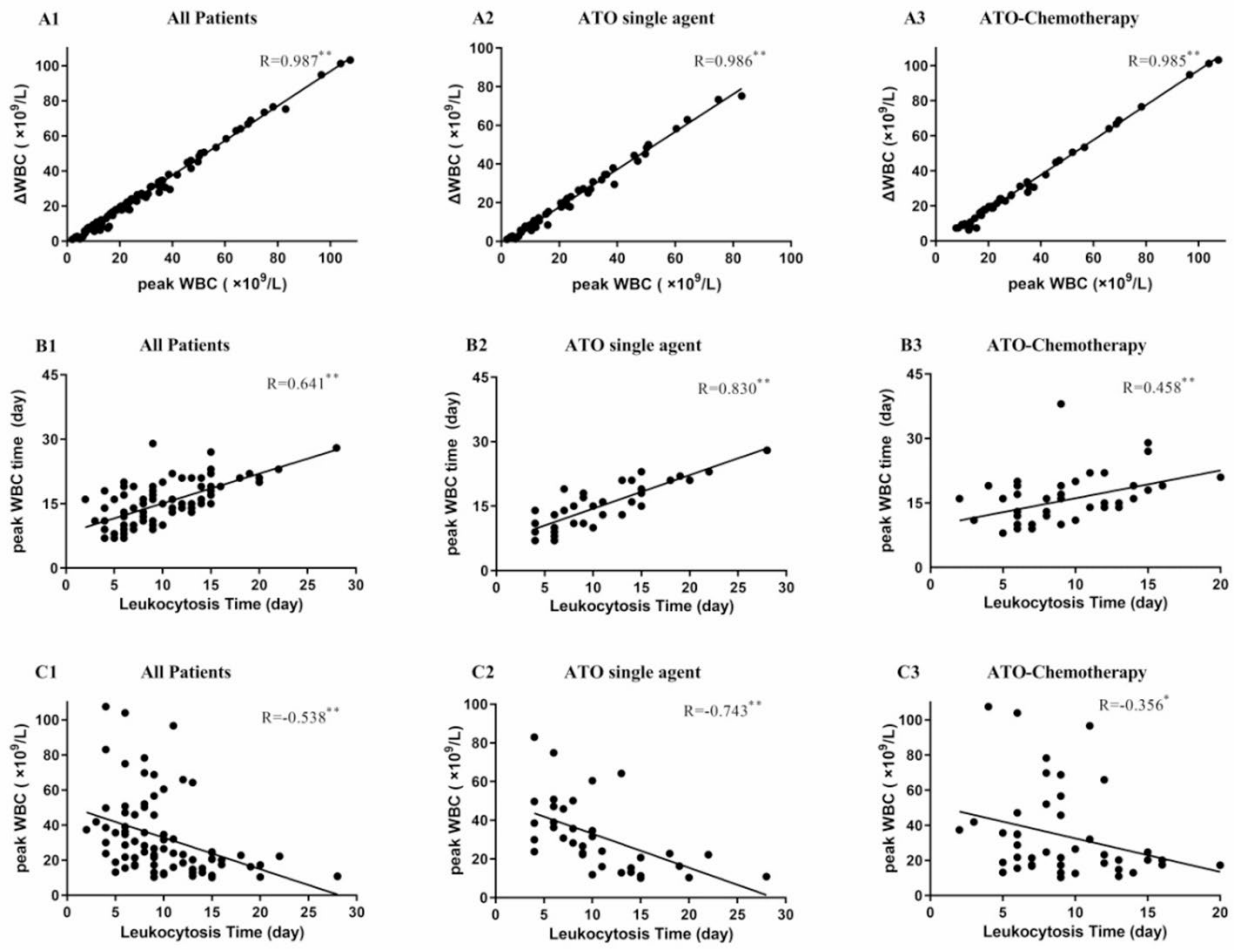
The correlations of leukocytosis indicators in APL patients. A1, A2 and A3 are correlations between peak WBC and ΔWBC; B1, B2 and B3 are correlations between peak WBC time (*the time to peak WBC*) and leukocytosis time (*the time to leukocytosis*); C1, C2 and C3 are correlations between *peak WBC* and *the time to leukocytosis*

A Spearman’s correlation coefficient analysis was applied to assess ΔWBC, peak WBC time, peak WBC and leukocytosis time. The results showed that there were positive correlations between ΔWBC and peak WBC in APL patients (*r* = 0.987, *r* = 0.986 and *r* = 0.985), and the above correlations were all considered statistically significant and highly correlated (*r* ≥ 0.8) (Fig. 5A). There were also positive Spearman’s correlations between peak WBC time and leukocytosis time. The correlation was moderately correlated in all patients (*r* = 0.641, 0.5 ≤ *r* < 0.8 ) and highly correlated in ATO single agent patients (*r* = 0.830, *r* ≥ 0.8). However, a low correlation existed in ATO-chemotherapy patients (*r* = 0.458, *r* < 0.5) (Fig. 5B). Between these indicators of *the peak WBC* and *the time to leukocytosis*, the difference in the negative correlation coefficient was large (*r*=-0.743) in ATO single agent patients than in ATO-chemotherapy patients (*r*=-0.356) (Fig. 5C).

### Univariate analysis of the predictors of ATO-induced leukocytosis

Basic clinical indicators or laboratory parameters were included in the univariate analysis of indicators of ATO-induced leukocytosis. The chi-square test (Fisher’s exact test for *n* < 5) was performed to compare leukocytosis and nonleukocytosis among all patients, ATO single agent patients and ATO-chemotherapy patients (Table 2).

**Table 2:** Univariate analysis of predictors of ATO-induced leukocytosis

Variables	All patients (*n* = 100)			ATO single agent patients (*n* = 51)			ATO-chemotherapy patients (*n* = 49)		
	Leukocytosis	Nonleukocytosis	*P*	Leukocytosis	Nonleukocytosis	*P*	Leukocytosis	Nonleukocytosis	*P*
Total	84	16		38	13		46	3	–
Age, years			0.540			0.949			1
≥ 50	25 (80.6%)	6 (19.4%)		15(75%)	5(25%)		10(90.9%)	1(9.1%)	
< 50	59(85.5%)	10 (14.5%)		23(74.2%)	8(25.8%)		36(94.7%)	2(5.3%)	
Sex			0.315			0.296			1
Female	41 (80.4%)	10 (19.6%)		17(68%)	8(32%)		24(92.3%)	2(27.7%)	
Male	43(87.8%)	6 (12.2%)		21(80.8%)	5(19.2%)		22(95.7%)	1(4.3%)	
Initial platelet count, ×10^9^/L			**0.028** ^*^			0.125			1
> 30	28 (73.7%)	10 (26.3%)		15(62.5%)	9(37.5%)		13(92.9%)	1(7.1%)	
≤ 30	56 (90.3%)	6 (9.7%)		23(85.2%)	4(14.8%)		33(94.3%)	2(5.7%)	
Initial hemoglobin level, g/L			0.419			0.472			1
> 80	38 (80.9%)	9(19.1%)		19(70.4%)	8(29.6%)		19 (95.0%)	1(5.0%)	
≤ 80	46 (86.8%)	7 (13.2%)		19(79.2%)	5(20.8%)		27(93.1%)	2(6.9%)	
Initial fibrinogen level, g/L			**0.005** ^**^			0.023^*^			0.288
≥ 1	56(77.8%)	16 (22.2%)		26(66.7%)	13(33.3%)		28(90.3%)	3(9.7%)	
< 1	28(100%)	0(0%)		12(100%)	0(0%)		18(100%)	0(0%)	
Induction therapy			**0.018** ^*^			-			-
Single ATO	38 (74.5%)	13(25.5%)		-	-		-	-	
ATO-chemotherapy	46(93.9%)	3 (6.1%)		-	-		-	-	
Initial WBC count, ×10^9^/L,			0.069			**0.048** ^*^			1
≤ 4	66(80.5%)	16(19.5%)		28(68.3%)	13(31.7%)		38(92.7%)	3(7.3%)	
> 4	18(100%)	0(0%)		10(100%)	0(0%)		8(100%)	0(0%)	
The time to double WBC, day			**0.001** ^**^			**0.012** ^*^			0.565
≤ 7th	51(96.2%)	2(3.8%)		20(95.2%)	1(4.8%)		31(96.9%)	1(3.1%)	
> 7th	33(70.2%)	14(29.8%)		18(60%)	12(40%)		15(88.2%)	2(11.8%)	
The time to peak WBC, day			**0.006** ^**^			**0.038** ^*^			0.242
≤ 2 W	39(97.5%)	1(2.5%)		17(94.4%)	1(5.6%)		22(100%)	0(0%)	
> 2 W	45(75%)	15(25%)		21(63.6%)	12(36.4%)		24(88.9%)	3(11.1%)	
ΔWBC, ×10^9^/L			**0.000** ^**^			**0.000** ^**^			**0.005** ^**^
≤ 10	12(42.9%)	16(57.1%)		6(34.6%)	13(65.4%)		6(66.7%)	3(33.3%)	
> 10	72(100%)	0(0%)		32(100%)	0(0%)		40(100%)	0(0%)	
Initial GGT			0.185			0.684			0.579
Normal	76(86.4%)	12(13.6%)		33(76.7%)	10(23.3%)		43(95.6%)	2(4.4%)	
Abnormal	8(66.7%)	4(33.3%)		5(62.5%)	3(37.5%)		3(75%)	1(25%)	
Initial AST			1			0.684			1
Normal	76(84.4%)	14(15.6%)		33(76.7%)	10(23.3%)		36(92.3%)	3(7.7%)	
Abnormal	8(80.0%)	2(20.0%)		5(62.5%)	3(37.5%)		10(100%)	0(0%)	
Initial ALT			0.925			0.442			1
Normal	67(84.8%)	12(15.2%)		32(78%)	9(22%)		35(92.1%)	3(7.9%)	
Abnormal	17(81.0%)	4(19.0%)		6(60%)	4(40%)		11(100%)	0(0%)	

Bold values and ^*^are statistically significant (P<0.05); and ^**^are statistically significant (P<0.01)

ALT: alanine aminotransferase; AST: aspartate aminotransferase; GGT: gamma-glutamyltransferase; WBC: white blood cell count; ΔWBC changes in WBC between the peak WBC and initial WBC before ATO treatment; ATO: arsenic trioxide

To explore predictors of leukocytosis, we divided the 100 patients into two groups based on whether the peak WBC count was higher than 10 × 10^9^/L. The leukocytosis rate was lower in ATO single agent patients than in ATO-chemotherapy patients (*P* = 0.018). The univariate analysis results showed that *initial platelet count*, *initial fibrinogen leve*l, *Induction therapy*, *the time to double WBC*, *the time to peak WBC* and ΔWBC were predictive factors for ATO-induced leukocytosis in all APL patients (all *P* < 0.05). The other clinical parameters were not significantly associated with leukocytosis in the univariate analysis (Table 2). Next, a chi-square test analysis for ATO-induced leukocytosis in ATO single agent patients and ATO-chemotherapy patients was performed. In the ATO single agent group, the analysis showed that the prognostic risk factors for ATO single agent patients were similar to those in all patients and the results showed that *initial fibrinogen level*, *initial WBC count*, *the time to double WBC*, *the time to peak WBC* and ΔWBC were predictors for leukocytosis (all *P* < 0.05). However, only ΔWBC (*P* = 0.005) was a risk factor for leukocytosis in ATO-chemotherapy patients (Table 2).

### Multivariate analysis of indicators for ATO-induced leukocytosis

In the univariable analysis, variables that were significant clinical factors associated with leukocytosis were further analyzed in a multivariate analysis model. Age, the time to double WBC, initial platelet count, initial GGT and induction therapy were included in the final model of APL patients models (Table 3).

**Table 3:** Multivariate analysis of risk factors for leukocytosis in APL patients during ATO-based induction therapy

Variables	Unfavorable Category	All Patients			ATO single agent Patients			ATO-chemotherapy		
		OR	95% CI	*P*	OR	95% CI	*P*	OR	95% CI	*P*
age	≥ 50	0.752	0.205–2.764	0.668	0.831	0.188–3.661	0.806	0.548	0.033–9.047	0.674
ATO single agent	No	0.430	0.094–1.964	0.276	-	-	-	-	-	-
The time to double WBC, day	≤ 7th	11.459	2.121–61.897	0.005^**^	16.603	1.635-168.635	0.018^*^	5.316	0.325–86.842	0.241
Initial platelet count, ×10^9^/L	≤ 30	0.297	0.079–1.125	0.074	0.317	0.072-1.400	0.129	0.217	0.009–5.114	0.343
Initial GGT	abnormal	3.881	0.78-19.354	0.098	3.267	0.453–23.570	0.240	9.553	0.396-230.185	0.164

Bold values and ^*^are statistically significant (P  < 0.05); ^**^ are statistically significant (P  < 0.01)

OR: odds ratio; CI: confidence interval; GGT: gamma-glutamyltransferase; WBC: white blood cell count; ATO: arsenic trioxide

Multivariate analysis was used to examine the effects of clinical indicators on leukocytosis. The same independent prognostic risk factors were *the time to double WBC ≤ 7th day* for leukocytosis in both all APL patients (OR = 11.459, *P* = 0.005) and ATO single agent patients (OR = 16.603, *P* = 0.018) during induction treatment progress (Table 3). Notably, the study revealed statistically significant inconsistencies across some risk factors for leukocytosis in ATO single agent patients and ATO-chemotherapy patients.

**Fig. 6:**
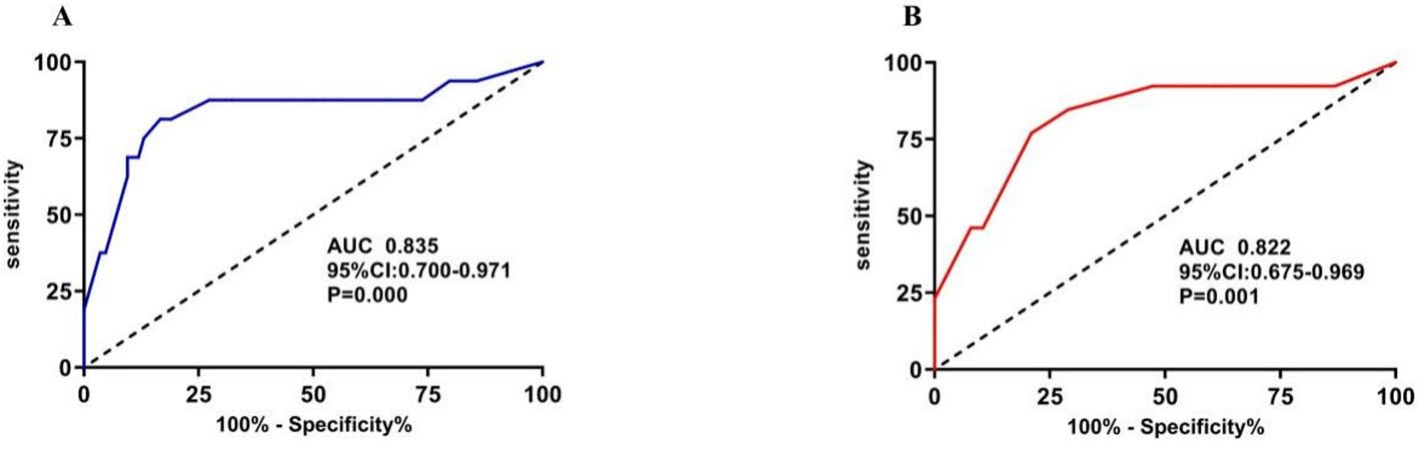
Receiver operating characteristic (ROC) curve for combined variables for leukocytosis. **A** Combined model for all APL patients (AUC = 0.835, 95% CI 0.700-0.971), including five variables from multivariate logistic regression: induction therapy type (β = -0.844), time to double WBC ≤7th day (β = +2.439), age ≥ 50 years (β = -0.285), initial platelet count ≤ 30 × 10⁹/L (β = -1.214), and initial GGT abnormal (β = +1.356). **B** Combined model for ATO single-agent patients (AUC = 0.822, 95% CI 0.675–0.969), including four variables: time to double WBC ≤7th day (β = +2.809), age ≥ 50 years (β = -0.185), initial platelet count ≤ 30 × 10⁹/L (β = -1.149), and initial GGT abnormal (β = +1.184). Full regression details including standard errors are provided in Supplementary Table S1. AUC, area under the ROC curve

To further explore whether the combined clinical indicators could be helpful in predicting the prognosis of patients with leukocytosis, we performed ROC analysis using the dichotomized variables and the AUC to quantify the predictive ability of the combined variable for leukocytosis. The AUC of the combined variable for leukocytosis in all APL patients was 0.835, and the ROC curve of the combined risk factors for ATO single agent leukocytosis had an AUC of 0.822 (Fig. 6). The Hosmer–Lemeshow test indicated that the model fit well for both leukocytosis in all APL patients and ATO single agent patient cohorts (χ^2^ = 14.016, df = 8, *P* = 0.081 and χ^2^ = 11.527, df = 7, *P* = 0.117, respectively). All these results indicate that the combined risk factors had considerable predictive value.

## Discussion

Leukocytosis is a common complication for APL patients during induction therapy and is different from that in other leukemias [23]. It has been shown that the distinction between reactive and neoplastic leukocytes is important for the treatment of leukemia [22]. The study of prognostic factors for leukocytosis in de novo patients treated with ATO-based therapy has scarcely been addressed in the reported study. The incidence of leukocytosis in APL patients that has been previously reported varies widely, ranging from 47% to 93.8% [1, 17, 24, 25]. An Italian study of 65 low to intermediate risk APL patients showed that the incidence of leukocytosis during induction therapy treated with ATRA-ATO was as high as 60%, with the peak WBC count occurring in the 2nd week after diagnosis [1]. The predictors of leukocytosis in that study included a lower platelet count and lower fibrinogen levels which were consistent with the results of our study. However, the results in our study are different from reported studies, and approximately 74.51% of de novo APL patients undergoing ATO single agent developed leukocytosis. Fewer studies have reported WBC kinetic features in APL patients undergoing ATO-based induction therapy. The different characteristics of WBC in this study may be related to the way of ATO infusion which continuous slow-rate infusion in ATO single agent patients can keep the drug stable at effective concentration. The lower incidence of leukocytosis in the ATO single-agent group (74.5% vs. 93.9%, *P* = 0.018) may be explained by several factors. Cytotoxic chemotherapy in the combination group may amplify leukocytosis through enhanced cytokine release and accelerated leukemic cell differentiation [26, 27]. Additionally, the continuous slow-rate infusion of ATO (> 18 h/day) in the single-agent group may result in a more gradual differentiation response compared with the combination group [28, 29]. These factors likely contributed to the observed difference in leukocytosis incidence between the two groups. The baseline WBC count for patients with leukocytosis was higher than that for patients who did not develop leukocytosis [24, 25] which is consistent with our findings (1.75 vs. 1.40 × 10^9^/L, *P* = 0.03). In our study, the median time to leukocytosis was the 9th day, which was earlier than in previous reported studies [24], suggesting that leukocytosis maybe associated with ATO treatment. The reported study of WBC proliferation indicators in 64 newly diagnosed APL patients found that *the time to peak WBC* was approximately the 10th day which was earlier than our results. and the time was earlier in patients who developed leukocytosis than in those who did not develop leukocytosis (15.00th vs. 23.00th day) [17]. *The peak WBC* in the reported research was higher than that in our study. This may be related to the differences in treatment regimen (the reported study was ATRA or ATRA + ATO, whereas ATO-based first-line treatment was used in our study). In addition, the patient population was heterogeneous, with non-high-risk acute promyelocytic leukemia patients in the reported study population and de novo APL patients with normal initial WBCs. *The time to double WBC* is an important independent factor in predicting peak WBC count, which is consistent with our study [17].

The difference in the effects of leukocytosis among reported studies could be a result of several factors. The development of leukocytosis is associated with induction therapy, especially differentiating agents such as ATRA and ATO [30]. The most likely explanation for differences relates to the unique cytokine release and leukocyte surface antigen. Previous studies have shown that serum G-CSF levels are positively correlated with WBC counts [31]. FLT3/ITD was associated with peripheral WBC count [32]; CD34 positivity was significantly correlated with WBC count [33]; CD2^+^ APL patients tended to have higher WBC counts than those with CD2-APL [34]. In addition, in our previous study, we found that the sulfhydryl level was associated with leukocytosis, and leukocytosis occurred earlier in APL patients treated with ATO in the low sulfhydryl level group [16]. Furthermore, the concurrent use of chemotherapy has probably determined differences in the incidence and severity of leukocytosis. In reported studies, there is an increased risk of early morbidity in APL patients with leukocytosis, mostly from bleeding22may be related to the powerful role of ATO in the apoptosis and autophagy of leukemia cells, as well as cellular differentiation [35]. This was inconsistent with the results in the reported study and may be related to the different data types and cutoff values of the dependent variables defined by the researchers.

As previously reported in the literature, WBC count ≥ 10 × 10^9^/L has been identified as an independent risk factor for early death in APL patients [36, 37], and leukocytosis has been associated with reduced complete remission rates and increased risk of hemorrhage and early mortality [38, 39]. These findings from the literature underscore the clinical importance of predicting and preventing leukocytosis during ATO-based induction therapy, which is the primary focus of the present study. ATO-induced leukocytosis may not represent an increase in leukemic cell proliferation, but rather a rapid increase in WBC counts resulting from a transient increase in the life span of leukemic cells, and a peak WBC count greater than 10 × 10^9^/L resulted in a significantly increased incidence of DS (20% vs. 59%) [40]. The prediction of leukocytosis is particularly important for the prevention of DS. In our study, the WBC growth rate in ATO single agent is faster than that in ATO-chemotherapy patients. We report several pretreatment variables predictive of the incidence of leukocytosis. Univariate logistic analysis was used to identify the associations between variables and leukocytosis. Multivariate logistic regression was used to determine the independent association between variables and leukocytosis. The odds ratio (OR) values *the time to double WBC* were high and presented good predictive abilities for both all patients and ATO single agent patients (OR 11.604 vs.16.603, respectively). In the analysis of the ROC curve for combined variables for leukocytosis, AUCs of > 0.8 were obtained in all patients and ATO single agent patients, the results suggested that the combined risk factors had considerable predictive value. The causes of the difference in indicators between the all patients and ATO single agent patient analyses may be related to the inconsistent mechanisms in chemotherapy and ATO. Interestingly, apart from *the time to double WBC≤7th day*, no other indicators contributed to leukocytosis development in APL patients during ATO-based induction therapy, and the mechanisms of ATO-induced leukocytosis deserve further research.

## Conclusions

The kinetics of leukocytosis induced by single-agent ATO and ATO-chemotherapy were different. And *the time to double WBC≤7th day* was identified as an independent poor prognostic factor for ATO-induced leukocytosis in APL patients.

## Supplementary Information

Below is the link to the electronic supplementary material.

Supplementary Material 1.

## Data Availability

The datasets used and/or analyzed during the current study are available from the corresponding author on reasonable request.

## Abbreviations

ATO: Arsenic trioxide (As2O3)
APL: Acute promyelocytic leukemia
ATRA: All-trans retinoic acid
ALT: Alanine aminotransferase
AST: Aspartate aminotransferase
AUC: Area under the ROC curve
CR: Complete remission
DS: Differentiation syndrome
GGT: Gamma-glutamyltransferase
ROC: Receiver operating characteristic
WBC: White blood cell count
ΔWBC: WBC changes between the peak WBC and initial WBC before ATO treatment
