# Supplementary material for: Predictors and kinetics of ATO-induced leukocytosis in acute promyelocytic leukemia patients: a retrospective study
Source: Discov Oncol. 2026 May 9;17:785. doi: 10.1007/s12672-026-05155-1 (PMC13201662; doi:10.1007/s12672-026-05155-1)
Supplement: Supplementary file 1 — Supplementary Material 1. [file 12672_2026_5155_MOESM1_ESM.docx]

**Supplementary Table S1.** Multivariate logistic regression models for predicting ATO-induced leukocytosis in APL patients: detailed regression coefficients, standard errors, and model performance metrics

| **Variable** | **β** | **SE** | **Wald** | **OR** | **95% CI** | ***P* value** |
| --- | --- | --- | --- | --- | --- | --- |
| Model A: All APL patients (n = 100) *\| AUC = 0.835 (95% CI 0.700–0.971) \| Hosmer–Lemeshow: χ² = 14.016, df = 8, P = 0.081* | | | | | | |
| Age (≥50 years) | −0.285 | 0.664 | 0.185 | 0.752 | 0.205–2.764 | 0.668 |
| Induction therapy (ATO single-agent) | −0.844 | 0.775 | 1.185 | 0.430 | 0.094–1.964 | 0.276 |
| Time to double WBC (≤7th day) | +2.439 | 0.861 | 8.030 | 11.459 | 2.121–61.897 | 0.005** |
| Initial platelet count (≤30×10⁹/L) | −1.214 | 0.678 | 3.210 | 0.297 | 0.079–1.125 | 0.074 |
| Initial GGT (abnormal) | +1.356 | 0.819 | 2.740 | 3.881 | 0.780–19.354 | 0.098 |
| Model B: ATO single-agent patients (n = 51) *\| AUC = 0.822 (95% CI 0.675–0.969) \| Hosmer–Lemeshow: χ² = 11.527, df = 7, P = 0.117* | | | | | | |
| Age (≥50 years) | −0.185 | 0.757 | 0.060 | 0.831 | 0.188–3.661 | 0.806 |
| Time to double WBC (≤7th day) | +2.810 | 1.183 | 5.644 | 16.603 | 1.635–168.635 | 0.018* |
| Initial platelet count (≤30×10⁹/L) | −1.149 | 0.757 | 2.303 | 0.317 | 0.072–1.400 | 0.129 |
| Initial GGT (abnormal) | +1.184 | 1.008 | 1.379 | 3.267 | 0.453–23.570 | 0.240 |
| Model C: ATO-chemotherapy patients (n = 49) *\| AUC = Not reported separately \| Hosmer–Lemeshow: —* | | | | | | |
| Age (≥50 years) | −0.602 | 1.432 | 0.176 | 0.548 | 0.033–9.047 | 0.674 |
| Time to double WBC (≤7th day) | +1.671 | 1.426 | 1.374 | 5.316 | 0.325–86.842 | 0.241 |
| Initial platelet count (≤30×10⁹/L) | −1.528 | 1.618 | 0.892 | 0.217 | 0.009–5.114 | 0.343 |
| Initial GGT (abnormal) | +2.257 | 1.624 | 1.932 | 9.553 | 0.396–230.185 | 0.164 |

**Abbreviations:** *β*, logistic regression coefficient; *SE*, standard error; *Wald*, Wald chi-square statistic; *OR*, odds ratio; *CI*, confidence interval; *AUC*, area under the receiver operating characteristic curve; *ATO*, arsenic trioxide; *APL*, acute promyelocytic leukemia; *WBC*, white blood cell count; *GGT*, gamma-glutamyltransferase.

**Statistical significance:** * P < 0.05; ** P < 0.01. Highlighted rows (yellow) indicate statistically significant independent predictors.

**Method note:** β coefficients and standard errors (SE) were derived from the multivariate logistic regression output using the relationships β = ln(OR) and SE = [ln(CI_upper_) − ln(CI_lower_)] ÷ 3.92, which are mathematically equivalent to direct SPSS output values. Wald = (β/SE)². The combined ROC models were constructed using logistic regression-derived predicted probabilities, which were imported into GraphPad Prism 9 for ROC curve analysis. Model goodness-of-fit was assessed using the Hosmer–Lemeshow test.

**Variable coding:** Age: 0 = < 50 years, 1 = ≥ 50 years; Induction therapy: 0 = ATO-chemotherapy, 1 = ATO single-agent; Time to double WBC: 0 = > 7th day, 1 = ≤ 7th day; Initial platelet: 0 = > 30×10⁹/L, 1 = ≤ 30×10⁹/L; Initial GGT: 0 = normal, 1 = abnormal.
